# Role of Bio-Based Polymers on Improving Turbulent Flow Characteristics: Materials and Application

**DOI:** 10.3390/polym9060209

**Published:** 2017-06-06

**Authors:** Wen Jiao Han, Hyoung Jin Choi

**Affiliations:** Department of Polymer Science and Engineering, Inha University, Incheon 22212, Korea; 22151728@inha.edu

**Keywords:** drag reduction, bio-polymer, turbulent flow, water-soluble

## Abstract

The remarkable ability of polymeric additives to reduce the level of frictional drag significantly in turbulent flow, even under extremely low dilutions, is known as turbulent drag-reduction behavior. Several bio-polymers have been assessed as promising drag-reducing agents for the potential replacement of high molecular weight synthetic polymers to improve safety and ameliorate environmental concerns. This article reviews the recent advances regarding the impact of several bio-polymer additives on turbulent drag reduction in either pipe or rotating disk flow systems, and their potential applications in the petroleum, biomedical, and agricultural industries.

## 1. Introduction

Bio-based polymers have attracted considerable attention in many areas [1], particularly due to the severe worldwide depletion of fossil fuel resources and increasing environmental pollution concerns. Therefore, huge efforts have been made on the production and application of biodegradable bio-based polymers as a substitute for traditional petroleum-based polymers [2]. Concurrently, the behaviors and material functions that animals and plants demonstrate have attracted great interest in terms of bio-inspired polymers because the natural world is filled with many interesting materials and phenomena that can be exploited in terms of bio-mimics. As one example, the mucus in the body surface of fish reduces frictional drag as they move through the water [3,4]. Rosen and Cornford [5] measured the drag reduction efficiency of fish mucus in turbulent flow, and revealed 65.9% drag reduction. Similar to the function of fish mucus, the addition of minute amounts of high molecular weight polymers into turbulent flow is considered a means of drag reduction [6]. Many water-soluble and oil-soluble long-chain polymers have efficient drag reduction capability.

Two typical types of flow occur depending on the shear viscosity, density, and velocity of a fluid flowing through a closed channel: laminar and turbulent flow. Generally, for laminar flow in a pipeline, all the fluid elements move along straight streamlines parallel to the flow direction with no eddies or lateral mixing [7]. In contrast, turbulent flow shows a less orderly flow region, in which eddies of the fluid elements can initiate strong lateral mixing. This behavior causes severe turbulent friction, resulting in considerable consumption of additional energy, which is a major problem in many industrial and engineering related areas, particularly for the transportation of liquid-like materials through strategic pipelines in the oil and gas industries. Turbulent flow formed during transportation in pipelines increases the frictional drag, dissipates pumping power, and reduces the transport efficiency [8]. Therefore, many different ways of reducing frictional drag in turbulent flow, such as the use of engineered surfaces, superhydrophobic surfaces, and additives as well as morphological alterations in the boundary layer flow, have been introduced for a wide range of engineering applications [9]. In particular, as a kind of biologically inspired surface, a superhydrophobic surface has attracted considerable interest as an effective choice for drag reduction. Gogte et al. [10] examined fluid flow over a hydrofoil with its surface randomly structured with sandpaper, and demonstrated appreciable drag reduction in the presence of surface texture combined with a superhydrophobic coating. Daniello et al. [11] also studied the turbulent channel flow over a micropatterned superhydrophobic surface and predicted that a superhydrophobic surface may provide an appreciable drag reduction mechanism for marine vessels. Compared to these passive control devices for drag reduction, the concept of drag reduction using drag-reducing agents in pipe flow has attracted considerable attention as an active controlling system with only the addition of minuscule amounts of polymeric additives. After the first report of the addition of minute amounts of poly(methyl methacrylate) molecules into the turbulent flow of monochlorobenzene [12], extensive research has been performed because of its significant scientific interest and wide range of engineering applications.

Generally, drag-reducing agents can be selected from insoluble materials, such as pulpwood, chopped nylon, and asbestos, as well as soluble materials, including polymers and surfactants [13]. Lee et al. [14], who examined potential ways of solving the problems of the mechanical degradation of polymeric additives, reported that fiber suspensions could exhibit drag reduction in turbulent pipe flow. On the other hand, in addition to their relatively low drag reduction efficiency, clogging or sedimentation is a significant problem in pipelines because these suspended solids are generally insoluble in the flow media. Compared to these insoluble additives, most studies focused on the use of high molecular weight polymers or cationic surfactants as drag-reducing agents in aqueous or hydrocarbon liquid media. Cationic surfactants can assemble reversibly to form a range of structures in aqueous solutions. These “self-repairable” characteristics, which occur after mechanically induced disassembly, have attracted considerable attention, particularly in circulating liquid applications [15]. On the other hand, polymeric additives strongly influence the behavior of turbulence flow owing to their particular rheological properties and chain flexibility under shear and extensional forces. Therefore, they have attracted considerable interest in engineering applications of turbulence manipulation, such as oil production and pipeline transportation [16,17], water heating and cooling systems [18,19], and agricultural field irrigation and biomedical applications [20,21]. Polymeric additives should have ultra-high molecular weight and good solubility in a liquid fluid medium. Minute amounts of polymeric drag-reducing agents can result in a particularly higher efficiency of drag reduction in both aqueous and organic liquids.

This review presents the recent progress in understanding the behavior of biopolymer-associated drag reduction in turbulent flow, with particular focus on the drag reduction of bio-based polymer additives in a turbulent flow. Non-biodegradable conventional synthetic polymers might have adverse environmental impacts and safety issues. Therefore, bio-polymers could be a promising alternative to synthetic polymeric additives. Regarding this concern, this review places special emphasis on the experimental parameters, mechanism analysis, and their applications.

## 2. Polymer-Associated Turbulent Drag Reduction Behavior

Despite the extensive investigations on scrutinizing the behavior of polymer-induced drag reduction over several decades, a complete understanding of its physical mechanism is lacking. 

Based on the Oldroyd’s theory of the wall effect [22], Toms [12] suggested that the shear thinning layer near the wall could reduce the friction factors. On the other hand, Walsh [23] examined polymeric molecules in solution using the Rouse model and reported that some shear thickening solutions also exhibit drag reduction ability.

The viscoelastic behavior of drag-reducing polymer solutions is an alternative mechanism of drag reduction. Attributing drag reduction to the two effects of viscoelasticity, Ruckenstein [24] used the Maxwell model as a constitutive equation and proposed that the instantaneous shear stress near the wall is smaller in a viscoelastic fluid than in a Newtonian fluid. In addition, he reported that the introduction of polymer additives can modify the near-wall turbulence structure. Metzner and Park [25] examined the turbulent flow characteristics of viscoelastic fluids by measuring the rheological properties of several viscoelastic polymer solutions. They revealed the pronounced suppression of turbulence and suggested a correlation between the degree of turbulence suppression observed and the ratio of the elastic to viscous forces. Renardy [26] also presented this mechanism, and suggested that the elongational viscosity is an important rheological property, possibly in combination with viscoelasticity. In contrast, Kostic [27] found that even at low polymer concentrations, the turbulence was suppressed considerably in the normal-to-main-flow direction, in which the solutions have no measurable elasticity. Therefore, he suggested that turbulence suppression, not elasticity, may be a determining factor in the drag reduction phenomena.

Another perspective was based on the molecular extension mechanism. Lumley [28] realized that polymer molecules could be expanded outside the viscous sublayer; thus, the thickness of the viscous sublayer increased, resulting in a decrease in the velocity gradient near the wall. Tulin [29] proposed that the drag reduction phenomena is related to the stretching of polymer molecules by the high strain rate in flow. At a high strain rate, the polymer chains tend to elongate along the principal strain rate axis, resulting in large extensions. In addition, as shear hardening occurs, bursts and sweeps at the wall layer flow are inhibited, thereby reducing the frictional drag. On the other hand, regarding vortex stretching, Gordon and Balakrishnan [30] explained the drag reduction phenomenon as a resistance to vortex stretching, which is caused by filament formation in a polymeric viscoelastic solution. Gadd [31] also suggested that larger polymer chains could affect the large eddies with lower stretching rates, which could lead to more rapid decay.

Tabor and de Gennes [32] proposed an elastic theory for drag reduction, and derived a kinetic and elastic energy transport equation for examining the energy transfer between the polymer and flow. Based on this theory, Sreenivasan and White [33] reported that drag reduction is due to the polymer molecules absorbing the small-scale turbulence energy into elastic energy, and preventing the turbulence cascade. Cadot et al. [34], however, reported the experimental results of drag reduction by polymeric additives in closed turbulent flow with a zero mean velocity at large Reynolds numbers. They used a smooth forcing scheme and a very rough forcing scheme, and proved that the wall effect is an important factor in polymer drag reduction. In addition, Virk [35] proposed an empirical correlation in that all dilute polymer systems for their turbulent drag reduction effect exhibit asymptotic behavior. Drag reduction begins only when the boundary layer becomes turbulent, and the capacity increases with increasing polymer concentration saturating beyond a certain degree. He suggested the existence of a maximum drag reduction, and the maximum level of achievable drag reduction appears to be limited by the Virk’s maximum drag reduction asymptote. This view has been accepted widely and used mainly in the velocity profile and friction diagram. Many studies have also derived Virk’s maximum drag reduction asymptote from theoretical considerations [36].

Other classical assumptions of the turbulent drag reduction mechanism include the molecular stretching of the polymers in the boundary layer. Macromolecules added to a turbulent solution increase the resistance to extensional flow, which is associated with shear hardening behavior, impeding turbulent bursts near the wall. Lumley [37] claimed that stretching of the random coiled polymeric chains increases the elongation viscosity, thereby suppressing turbulence fluctuations and resulting in a decrease in wall friction by a thickening of the buffer layer. On the other hand, Min et al. [38] suggested that polymeric additives in turbulent flow store the elastic energy from the flow near the wall, and when the relaxation time is long enough, this high elastic energy is transported to and dissipated into the buffer by near-wall vortical motion, resulting in the significant polymer-induced turbulent drag reduction.

The non-isotropic properties were also proposed to explain the drag reduction phenomenon. The polymer chains migrate in the solution affected by shear flow, resulting in a change in the structure and viscosity of the solution. As the shear rate is directional, the structure and viscosity of the solution will be non-isotropic. This mechanism is based on the shear rate dependence of the drag reduction fluid [39].

A range of extra mechanisms have been proposed to explain the drag reduction phenomena, and more studies of flow systems and drag-reducing agents will be helpful in analyzing these complex drag reduction mechanisms.

## 3. Flow Geometry for Turbulence

**Figure 1:**
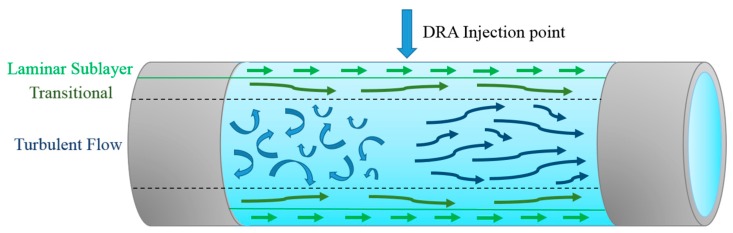
Schematic diagram for the methods of added polymer to a solution with pipeline turbulent flow.

To study the drag reduction properties, most studies adopted turbulent pipe flow that produces pressure-driven flow within a closed conduit as an internal flow. As shown in Figure 1, there are three flow layers in turbulent pipe flow, including a laminar sublayer (next to the wall), a transitional zone, and a fully turbulent layer (in the center of pipe). With the addition of polymer additives to a pipeline flow system, minute amounts of polymer suppress the formation of turbulent bursts in the buffer region and the propagation of turbulent eddies [40].

In pipe flow, the drag reduction factors are defined by the following equation:

(1) $$DR(\%)=(\frac{f_{s}-f_{a}}{f_{s}})\times 100=(\frac{\Delta P_{s}-\Delta P_{a}}{\Delta P_{s}})\times 100$$

where *f_s_* and *f_a_* describe the friction factor values, and Δ*P_s_* and Δ*P_a_* represent the pressure gradients for a clear solvent and solution with additives, respectively.

A high-precision rotating disk-type apparatus (RDA) was introduced to measure the drag reduction efficiency. Choi and Jhon [41] adopted the RDA system to measure both the frictional reduction and mechanical shear degradation of polymeric materials. Compared to pipe flow, which produces pressure driven flow within a closed conduit, the shear induced rotating disk flow is a drag and external flow with no-imposed pressure gradients and a different origin of the turbulent boundary layer. The turbulence is produced by driving a disk surface located within the fluid. The flow in the neighborhood of the rotating disk is of practical importance, particularly in connection with rotary machines. In addition, the rotating disk can reduce turbulent drag by itself if placed underneath fully developed turbulent flow [42,43,44]. Dickerson et al. [45] examined the polymeric fluid drag reduction using a high Reynolds number rotating disk system.

In this RDA system, the rotational Reynolds number (*N*_Re_), which is a dimensionless quantity that represents the ratio of the inertial force to cohesive force, can be defined by the following: solution density, ρ; viscosity, μ; disk radius, *r*; and angular velocity of this rotational disks, ω. This is expressed in the following relationship [46]:

(2) NRe=ρr2ωμ

The drag reduction efficiency %*DR* can be calculated as follows:

(3) $$\%DR\;=(\frac{T_{S}-T_{P}}{T_{S}})\times 100$$

where *T_S_* is the torque measured in a pure solvent, and *T_P_* is the torque measured in a polymer solution with a fixed disk rotation speed. On the other hand, there is a difference in the turbulent drag reduction from the internal flow, such as pipe flow, and external flow, such as the RDA. While frictional drag is obtained for internal flow, the total drag, which is a combination of frictional drag and form drag, is obtained for external flow because in the case of external flow, both flow over flat plates and flow around submerged objects are coupled. Owing to this difference in their turbulent drag reduction efficiencies, a maximum of 80% of the turbulent drag reduction can be obtained in tube flow, while the RDA generally produces approximately 50% of the maximum drag reduction [46]. 

Recently, Sreedhar et al. [47] adopted two different experimental setups of a gravity-driven method and pipe friction method to measure the drag reduction of hydroxypropyl methylcellulose, polyacrylamide, and polyethylene glycol. The gravity-driven method, with Reynolds numbers ranging from 15,000 to 35,000, was used to measure the efflux time required for the discharge of a certain volume of fluid through a given diameter pipe under the action of gravity. In this case, the efflux time is inversely proportional to the flow rate, and the formula for drag reduction can be calculated as follows:

(4) $$\frac{Q_{w}}{Q_{p}}=\frac{Area\times Height/t_{w}}{Area\times Height/t_{p}}$$

(5) $$\%DR=\left(1-\frac{Q_{w}}{Q_{p}}\right)\times 100=\left(1-\frac{t_{p}}{t_{w}}\right)\times 100$$

where *Q_w_*, *t_w_* and *Q_p_*, *t_p_*, are the flow rate and efflux time for the water and polymer solution, respectively.

In the pipe friction apparatus with Reynolds numbers ranging from 50,000 to 75,000, the pressure drops for both pure water and polymer solution are determined using differential pressure sensors fitted to the pipe. In this case, the pressure difference is varied with different concentrations of the polymer. The friction coefficient is proportional to the pressure difference, and the friction factor becomes the key parameter for evaluating the drag reduction, as shown in the following Equations (6) and (7);

(6) $$f=\frac{\left(△P\right)r}{\rho V^{2}L}$$

(7) $$\%DR=\left(1-\frac{△P_{p}}{△P_{w}}\;\right)\times 100=\left(1-\frac{f_{p}}{f_{w}}\right)\times 100$$

where Δ*P* is the pressure difference, *f* is the friction factor, and *V* is the velocity. The following are constant: radius, *r*; pipe length, *L*; and density, ρ

Although the pipe friction apparatus can achieve a higher flow rate than the gravity-driven method, it is difficult to eliminate the interference of the drive device, which is not conducive to improving the flow quality of the flow field and reducing the turbulence of the flow field. The gravitational method is the flow system of the potential energy of a fluid to the dynamic potential energy, which could avoid interference of the driving device to the flow field [48].

Burnishev et al. [49] chose inertially driven von Karman swirling flow between two counter-rotating bladed disks system instead of conventional pipe flow. Compared to linearly stable pipe flow, the transition of a fully developed turbulent regime is better defined; it is easier to control this closed flow and track the mechanical chopping of the polymeric molecules. In addition, the Taylor–Couette device [50] and two-dimensional flow, such as a soap film, have also been adopted for drag reduction.

## 4. Drag-Reducing Bio-Based Polymers

Bio-based polymers are polymeric biomolecules that are produced by living organisms. Polysaccharides are an important class of biopolymers that have attracted considerable research attention [51]. Polysaccharides comprise multiple monosaccharide units bound together by glycosidic linkages to form large, linear or highly branched polymers. They are used as raw materials for a range of applications because of their safety, non-toxicity, low cost, biodegradability, biocompatibility, and reproducibility [52]. This unique multi-functionality have led to their widespread use as thickeners, emulsifiers, suspending agents, and moisturizers in the food, cosmetics, pharmaceuticals, and paint industries. Among them, many studies have also identified polysaccharides as natural turbulent drag-reducing agents. On the other hand, the biodegradability of polysaccharides may reduce the shelf life and efficiency period of polymer. To overcome this, several attempts, such as derivatization, cross-linking, and grafting, have been made to make their properties more suitable for applications [53,54,55].

This review paper focuses on xanthan gum, guar gum, DNA, amylopectin, cellulose, carrageenan, okra mucilage, and other plant mucilage, on their drag reduction capabilities and applications. Note that recent turbulent drag-reducing water-soluble polymers are also covered, focusing on their applications [56]. 

### 4.1. Natural Gum Family

Natural gums are polysaccharides that can be obtained from wood elements of plants or seed coatings. Because trace amounts of natural gums can increase the viscosity of an aqueous solution considerably, they are used widely as thickeners, binders, flocculants, and stabilizers, etc., in various industries.

Among them, xanthan gum (XG), which is a complex microbial extracellular polysaccharide produced by fermentation by the bacterium xanthomonas campestris, is best well-known as an effective stabilizer for water-based systems. As an acidic polymer, its structure is comprised of a linear main chain of (1 → 4)-β-d-glucose, forming a backbone with trisaccharide side-chains on every second d-glucose [57,58].

Owing to its superior rheological properties, xanthan gum is used widely in many industrial areas. The typical agriculture applications of xanthan gum are used to improve the flow ability in insecticides, herbicides, and fungicides [59]. In the food industry, xanthan gum has been used as a viscosity enhancing agent. Xanthan gum can also form high viscosity solutions at low shear forces, which make it useful in the petroleum industry, including oil drilling fluids, fracturing, pipeline cleaning, and enhanced oil recovery.

**Figure 2:**
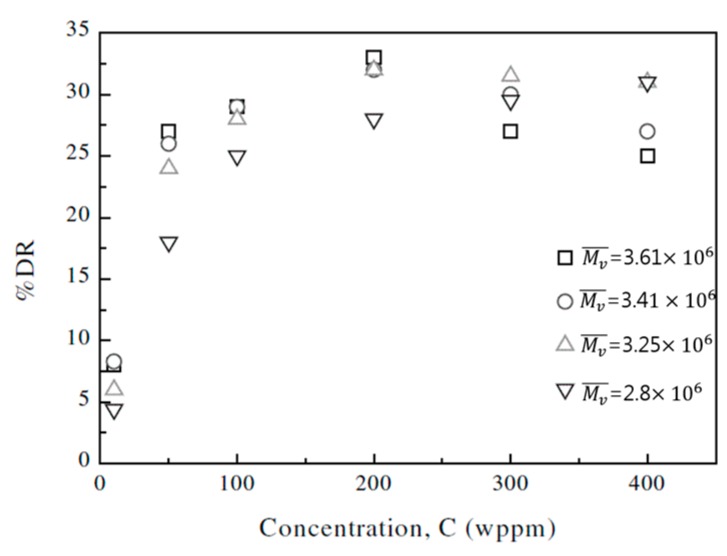
Initial %*DR* versus the xanthan gum concentration at four different molecular weights at 1800 rpm, reprinted with permission from [61]. Copyright Elsevier Science Ltd., 2001.

Kim et al. [60] examined the effects of the rod-like polysaccharide xanthan gum concentration on drag reduction using a rotating disk apparatus and its drag-reducing efficiency was analyzed using a three-parameter empirical relationship between the drag reduction and concentration, which was proposed by Choi and Jhon [41]. They reported that polysaccharide xanthan gum is a more shear-stable drag-reduction agent than most flexible polymers. Sohn et al. [61] examined the effects of the molecular parameters on the drag reduction of xanthan gum, including the polymer concentration, polymer molecular weight, rotational disk speed, temperature and ionic strength of the solution. Figure 2 shows the initial drag reduction efficiencies versus polymer concentration at different molecular weights. They reported that the drag-reduction efficiency of xanthan gum is closely related to the molecular parameters ($\bar{M_{v}}$) and it behaves as a more shear-stable drag-reducing agent in deionized water and salt solutions, than most flexible polymers.

Pereira et al. [62] compared the drag-reduction efficiency of poly(ethylene oxide) (PEO), polyacrylamide (PAAM), and xanthan gum over time using a rotating cylindrical double gap device. As a rigid polymer, the relative drag reduction of a xanthan gum solution was affected weakly by the Reynolds number, which is different from flexible polymers. On the other hand, the structure of xanthan gum is strongly dependent on the temperature compared to flexible polymers. At moderate temperatures, xanthan gum exhibits a stable methodic helical conformation, resulting in a rigid molecular structure [63,64]. For the concentration test, a solution of XG is strongly affected by pre-shearing, indicating the presence of polymer aggregates, and degradation does not occur in xanthan gum solutions.

**Figure 3:**
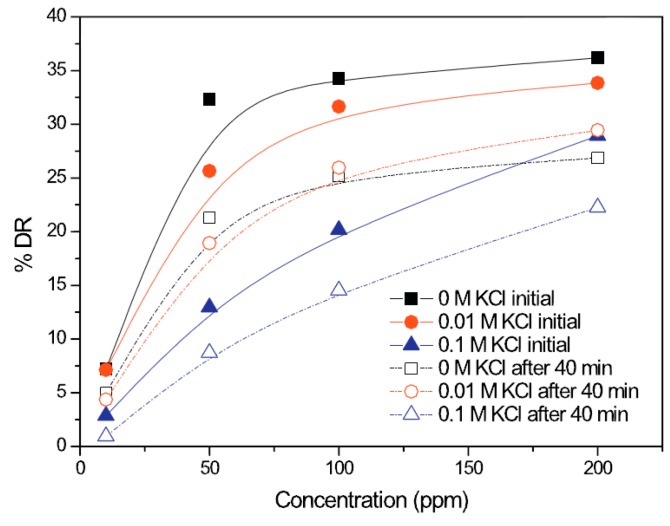
Concentration dependence of %*DR* with xanthan gum (XG), reprinted with permission from [65]. Copyright Elsevier Ltd., 2015.

**Figure 4:**
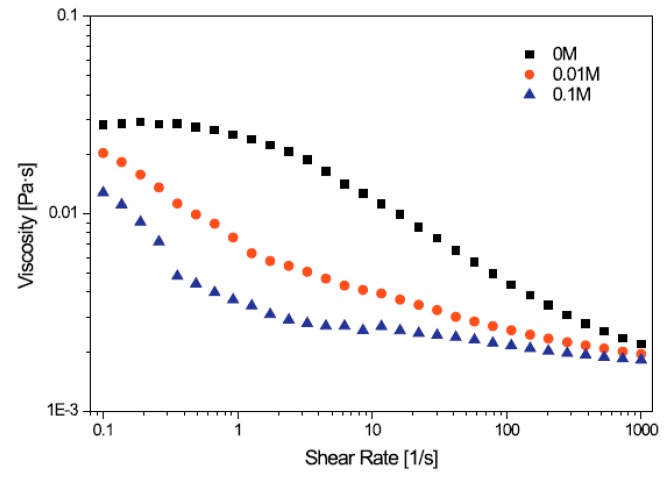
Shear viscosity as a function of various concentrations of KCl at 200 ppm, reprinted with permission from [65]. Copyright Elsevier Ltd., 2015.

Recently, Hong et al. [65] examined the turbulent drag reduction efficiency induced by a small amount of xanthan gum in aqueous KCl solutions with different concentrations using a rotating disc in a closed chamber. A comparison of the drag reduction efficiencies of an initial value and the one after 40 min showed that the mechanical degradation of XG becomes less severe at a higher KCl concentration. The added salt ions interact with the anionic charge of xanthan gum, which can induce conformation changes of the xanthan gum in solution and lead to changes in shear viscosity. Figure 3 shows the drag reduction as a function of the xanthan gum concentration initially and after 40 min. The drag reduction was increased at higher xanthan gum concentrations and the drag reduction efficiencies of XG/KCl became lower at higher KCl concentrations. The polymer chain conformation of xanthan gum becomes more rigid, resulting in lower sensitivity to high shear conditions. The molecular conformation of XG/KCl solutions was analyzed according to the rheological properties. Figure 4 presents the shear viscosity as a function of the various KCl concentrations for 200 ppm xanthan gum; the viscosity of the salt solutions increased with decreasing KCl concentration. With the addition of salt, the chains of xanthan gum tend to adopt a more compact helical backbone conformation due to electrostatic interactions. The shear viscosity of a XG/KCl solution decreases with decreasing hydrodynamic volume. 

On the other hand, regarding the shear-thinning behavior of the drag-reducing polymer solution, Malik and Mashelkar [66] also observed the shear rate dependent shear viscosity of hydrogen bonding mediated interpolymer complex based drag reducer in a wide range of shear rate.

To apply those experimental concepts to the design of a polymer-induced drag reduction piping system, Campolo et al. [67] ran a series of drag reduction experiments using a semi rigid bio-polymeric xanthan gum in a large diameter pipe. Through cost-effectiveness analysis, they found sets of operating conditions for the profitable use of xanthan gum as DRA, including an analysis of the *DR%* data under different conditions and the value of the cost of combining the energy/polymer prices with the pipeline characteristics. Furthermore, they also observed that both the increase of shear viscosity and shear-thinning behavior for increased xanthan gum concentrations in a turbulent flow condition [67].

On the other hand, guar gum (GG), which is a type of galactomannan, can be obtained from the seeds of the Cyamopsis tetragonolobus [68]. Owing to the long chain molecular structure and the abundance of hydroxyl groups across the chain, hydrogen bonds form in an aqueous solution of guar gum, which imparts significant thickening and viscosity to the solution [69]. Its unique properties, such as rapid dissolution in cold water, thickening, emulsifying, wide pH stability, biodegradability, etc., make it suitable for many applications, including hydraulic fracturing, food, agriculture, paper, cosmetics, bioremediation, and pharmaceuticals.

**Figure 5:**
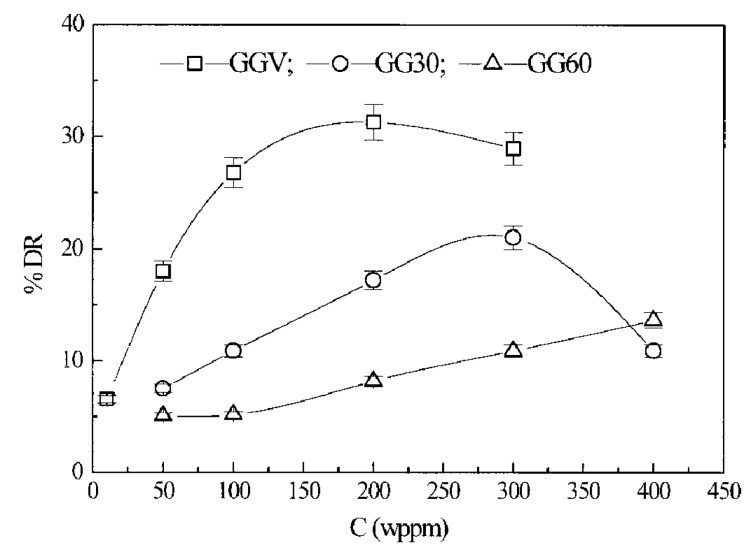
%*DR* versus the GG concentration for three different molecular weights, reprinted with permission from [70]. Copyright John Wiley and Sons, 2002.

Kim et al. [70] compared the turbulent drag reduction characteristics of guar gum in water using a rotating disk apparatus. Through an ultrasonic degradation method, the different molecular weight fractions of guar gum, including GGV (virgin), GG30 (ultrasonicated for 30 min), and GG60 (ultrasonicated for 60 min) were obtained. Figure 5 presents the results of the drag reduction efficiency versus polymer concentration. They suggested that guar gum is a useful, water-soluble drag reducer that is more stable to mechanical stress than the synthetic water-soluble drag reducer.

Hong et al. [71] examined the mechanical degradation of three different guar gums. They adopted the stretched-exponential model to describe the time-dependent mechanical degradation of guar gums, which was superior to other degradation models, such as the stretched-exponential model and single relaxation process.

The intrinsic structure and properties of guar gum make it suitable for applications in many areas, but it also has some drawbacks. The chemical modification of guar gum not only overcomes its inherent deficiencies by introducing new functional groups, it also maintains the advantages of guar gum to the greatest degree [72]. 

To solve the biodegradability of guar gum, Deshmukh et al. [73] prepared graft copolymers of guar gum and PAAM (Gm1–Gm3) and compared them with commercial (CGG) and purified guar gum (PGG). The results showed that purification and grafting significantly enhances the drag reduction effectiveness and biodegradation resistance of guar gum. Similarly, Deshmukh et al. [74] assessed seven graft copolymers of guar gum and PAM based on their drag reduction characteristics, shear stability, and biodegradation rate. They found that the drag-reduction characteristics could be enhanced by increasing the number of longer grafts in the molecule. The biodegradation could be minimized through this graft copolymerization in that there was no biodegradation observed for up to 10 days.

Behari et al. [75] also examined the grafting of methacrylamide onto guar gum using a suitable redox pair of potassium chromate/malonic acid, and studied the reaction conditions that could give more graft copolymer and produce less homopolymer. They reported that the grafting parameters increased with increasing chromate ion or hydrogen ion concentration. 

**Figure 6:**
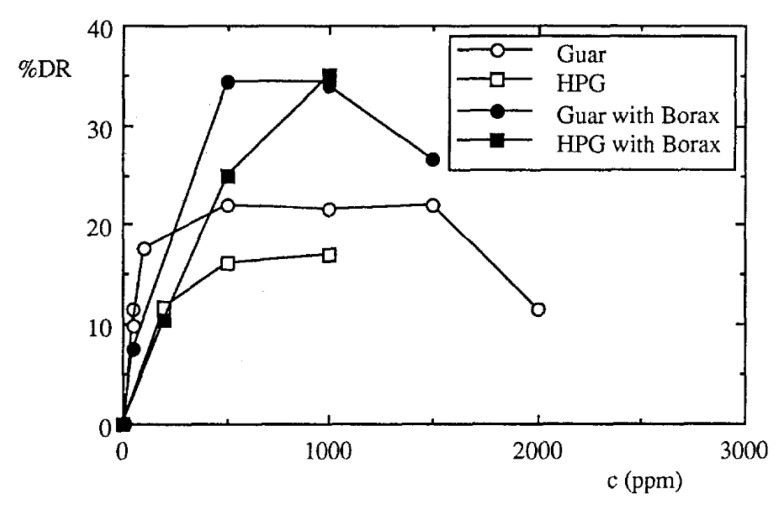
Drag reduction percentage at *v* = 2.2 m/s, reprinted with permission from [76]. Copyright Springer, 1996.

In addition to grafting, cross-linking is the other method for chemical modifications of guar gum. Bello et al. [76] examined the effects of borax as a cross-linking agent on the drag reduction ability of guar gum solutions using horizontal pipes. The presence of intermolecular cross links increased the size of the macromolecules and their apparent molecular weight. On the other hand, cross-linking had no effect on the degradation rate of the solution. The drag reduction efficiencies of gum with and without borax were compared, as shown in Figure 6. This shows that the addition of a cross-linking agent to a guar gum solution with a concentration below the degree of gelation results in an up to 35% increase in the drag reduction percentage.

In contrast, Phukan et al. [77] examined the application of drag-reducing guar gum in a sprinkler irrigation system. The drag-reduction effect, radius of coverage, and infiltration rate of commercial and purified guar gum were compared. They found that guar gum after purification shows better performance than that before in all cases. The maximum drag reductions achieved for both 500 ppm of purified guar gum and 1000 ppm of commercial guar gum were 40%.

### 4.2. λ-DNA

DNA is a long chain length polymer made of repeating units called nucleotides and its structure is non-static. Hand and Williams [78] conducted a test using calf-thymus DNA with pH control and reported that DNA in the natural state is preferable to the random-coil conformation for maximum drag reduction.

**Figure 7:**
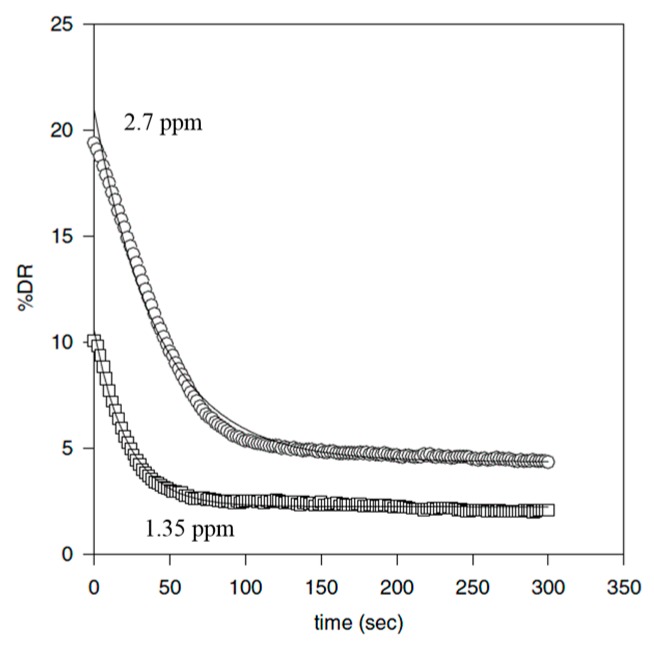
Drag reduction versus time with λ-DNA at different concentrations, reprinted with permission from [79]. Copyright American Physical Society, 2002.

**Figure 8:**
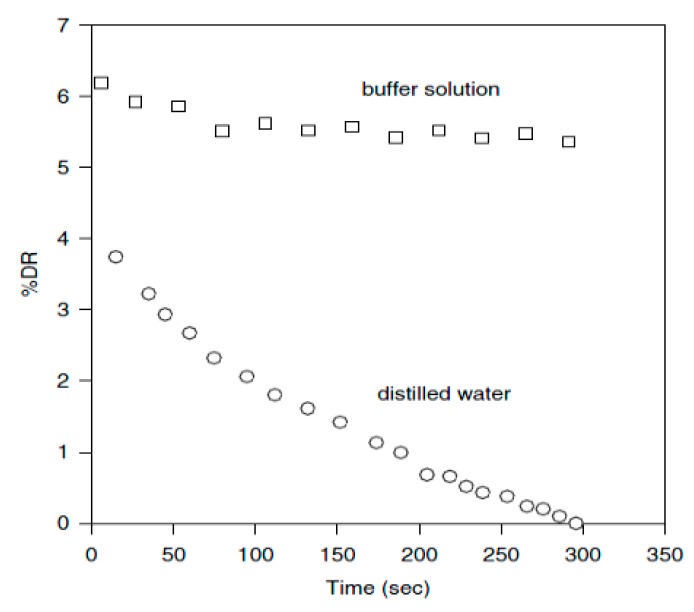
Difference between measured %*DR* of 1.35 ppm λ-DNA at *R_e_* = 7 × 10^5^ in a buffer solution and distilled water, reprinted with permission from [79]. Copyright American Physical Society, 2002.

Compared to synthetic high molecular weight linear chain polymers, DNA has been studied as a drag-reducing agent because of its high molecular weight and unique configurational change. Choi et al. [79] adopted λ-DNA molecules to probe the mechanism of the drag-reduction efficiency and mechanical molecular degradation. They reported that λ-DNA is a better drag reducer than the traditional linear polymer of PEO. Figure 7 presents the drag-reduction efficiency versus time with λ-DNA. For the mechanical degradation test, the mechanism of λ-DNA degradation is completely different with PEO, which is always being cut in half. Figure 8 shows the different drag reduction percentage for 1.35 ppm λ-DNA in distilled water and buffer solution, respectively. The λ-DNA in distilled water can be denatured from a double-stranded natural state to two single-strand molecules, which exhibit similar behavior to regular linear polymers. This result also shows that prior to degradation, the drag reducing power of λ-DNA in a buffer solution was approximately twice that in distilled water. This suggests that double-stranded DNA is a better drag-reducing agent than single-stranded DNA, which means that double-stranded DNA not only produces a higher drag reduction efficiency at the same concentration, but also has greater resistance to degradation.

To investigate the DNA degradation mechanism, Lim et al. [80] examined the turbulent drag reduction of λ-DNA, and compared the data with PAAM using a continuous and stepwise mode via a self-designed rotating disk apparatus. They confirmed Choi et al.’s [79] discovery of the half-length degradation of λ-DNA and showed that the resistance of helically stranded λ-DNA is stronger than PAAM. 

Wagner et al. [81] reported the turbulent drag reduction of polyelectrolyte solutions, including DNA and hydrolyzed PAM with salt concentration control and reported that the drag reduction efficiencies increased with increasing flexibility. In addition, they reported that the elongation viscosity is a relevant macroscopic quantity that describes the ability of polymers to cause drag reduction.

Similarly, Bonn et al. [82] examined the reduction of turbulent energy dissipation by adding biopolymer DNA. They also confirmed that the elongation viscosities of polymer solutions are related directly to the turbulent drag reduction.

**Figure 9:**
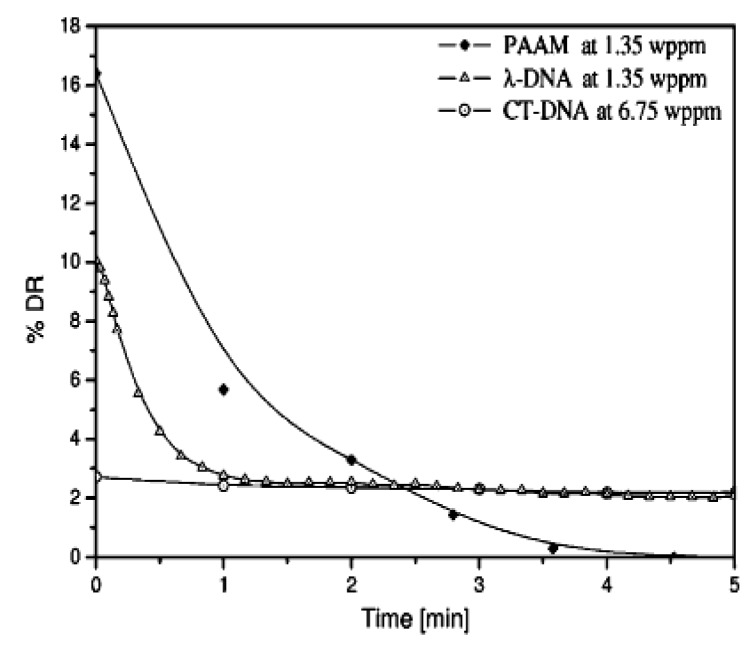
Comparison of the drag reducing characteristics of calf thymus DNA (CT-DNA) with those of linear high molecular weight synthetic polyacrylamide (PAAM) (polydisperse) and monodisperse λ-DNA, reprinted with permission from [83]. Copyright Elsevier, 2005.

**Figure 10:**
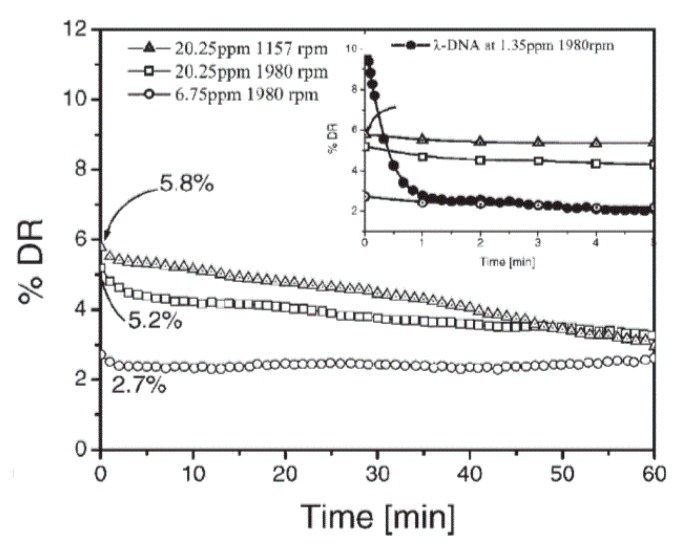
DR efficiency of CT-DNA for different concentrations and RPM (revolutions per minute). The inset shows a comparison of the initial drag reducing efficiency of CT-DNA with λ-DNA, reprinted with permission from [84]. Copyright John Wiley and Sons, 2007.

On the other hand, calf thymus DNA (CT-DNA), as a potential candidate, is also used for drag reduction studies. Lim et al. [83,84] assessed the durability of calf thymus DNA (CT-DNA) and compared the results with those of λ-DNA and linear polymer PAAM. Although CT-DNA possesses a higher molecular weight, almost no drag reduction efficiency was observed compared to λ-DNA, as shown in Figure 9 and Figure 10. This is because most of the chains of CT-DNA showed major degradation within seconds. On the other hand, although PAAM showed a higher drag reduction efficiency, this effect diminished to zero within 5 min. Therefore, DNA possesses much stronger resistance in turbulent flow owing to its double-helix structure.

### 4.3. Amylopectin

Amylopectin is a soluble polysaccharide and a highly branched polymer with a high molecular weight. This is one of the constituents of starch, the other being amylase, which is a linear low molecular weight polymer. Starch and its component amylopectin have been evaluated extensively as efficient flocculating agents and turbulent drag reducers for a range of industrial applications.

Stelter et al. [85] compared the flocculating and turbulent drag-reducing efficiency of grafted and ungrafted amylopectin with PAM focusing on shear and extensional investigations. The grafted amylopectin exhibited enhanced flocculation, turbulent drag reduction, and viscosifying attributes. Grafted amylopectin becomes semi-flexible due to ring opening and added flexible PAM chains.

Wunderlich et al. [86] also conducted similar research on grafted and ungrafted polysaccharides, including CMC, guar gum, sodium alginate and starch, and the same conclusions that the grafted polysaccharide solution exhibits viscosifying attributes were confirmed.

**Figure 11:**
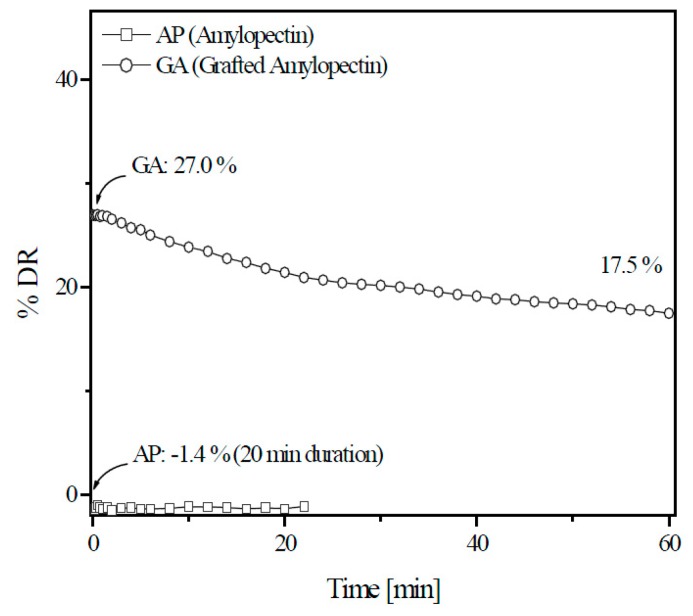
Percentage drag reduction versus time for amylopectin and its derivative (GA) at 25 °C, 1740 rpm (*N*_Re_ = 1.0 × 10^6^), and 30 wppm, reprinted with permission from [87]. Copyright De Gruyter, 2013.

To examine the drag reduction efficiencies and shear stabilities of amylopectin and its derivative, Lim et al. [87] examined the drag reduction efficiency using a rotating disk apparatus by changing factors, such as temperature, rotation speed, and polymer concentration. The derivative was synthesized by introducing PAAM branches into the amylopectin backbone and the morphology was changed from particles to lumps. Figure 11 shows that compared to amylopectin, which has no noticeable drag-reducing effect with 30 ppm, the same amount of grafted derivative shows relatively high initial drag reduction efficiencies (27%) and strong shear resistance.

### 4.4. Cellulose

Cellulose, as a structural component in the cell walls of plants, is one of the most common polysaccharides. The polymer is composed of d-glucose, which condenses through several hundred to many thousands of β (1 → 4)-glycosidic bonds. Cellulose is insoluble in both aqueous and organic solvents due to its strong hydrogen bonding, which limits its application in industry. Therefore, many researchers have examined the chemical modification of cellulose. Hydroxyethyl cellulose (HEC) and carboxymethyl cellulose (CMC) are the two types of cellulose derivatives that are commonly used in drag reduction. Both possess good solubility in water, and are also applied to other fields, such as paper, foods, flocculation, and cosmetics.

**Figure 12:**
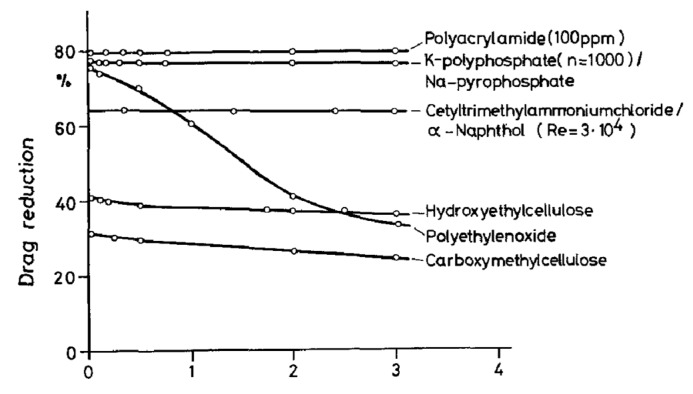
Effectiveness of various flow enhancers at a concentration of 300 ppm in water as a function of the circulation time at *Re* = 10^5^ and *d* = 14 mm, reprinted with permission from [88]. Copyright Springer, 1985.

Interthal and Wilski [88] examined a range of substances for their suitability as drag-reducing agents both in the laboratory and in various industrial pipelines. The effects of concentration, temperature, solvent, and pH on the experimental results were also studied. Figure 12 presents the effectiveness of various drag-reducing agents with a 300 ppm concentration at *R_e_* = 10^5^ in pipe flow. Both HEC and CMC exhibited notable drag reduction efficiency with satisfactory stability to mechanical degradation. The maximum drag reductions of HEC and CMC with 14 mm diameter pipes were 42% and 32%, respectively.

**Figure 13:**
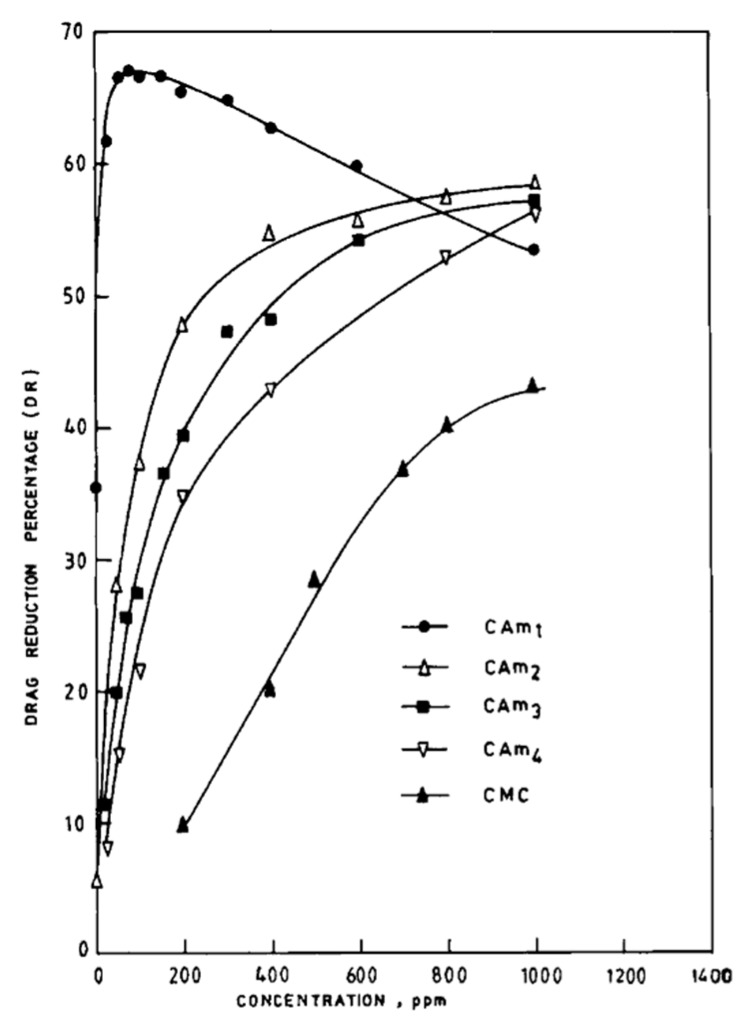
Drag reduction versus polymer concentration for carboxymethyl cellulose (CMC) and its graft copolymers, reprinted with permission from [89]. Copyright John Wiley and Sons, 1991.

Singh et al. [89] synthesized CMC-based graft copolymers by grafting acrylamide chains onto their backbones, and examined their drag reduction efficacy, rheological property, and biodegradability. The presence of grafted polyacrylamide (PAAM) chains resulted in enhanced drag reduction effects, good shear stability, and significant biodegradable insensitivity, in which these factors were found to depend on the number and length of the grafts. Figure 13 shows the drag-reduction efficacies of unmodified CMC and graft copolymers. All the graft copolymers exhibited higher efficiency than the unmodified CMC, and a maximum drag reduction of approximately 68% was obtained at a CAM-1 concentration of up to 75 ppm. Using the synthesized CMC-*g*-PAAM copolymers, Biswal and Singh [90] also reported that these grafted copolymers have conspicuous flocculation and viscosifying characteristics.

### 4.5. Carrageenan

Carrageenan, a type of linear sulphated polysaccharide, is extracted from red edible seaweed. The polysaccharide is divided into three main varieties according to the degree of sulphation: κ-carrageenan, ι-carrageenan and λ-carrageenan. Carrageenan and its derivatives are used widely in the food, personal-care and biomedicine industries as stabilizing, gelling, and thickening agents.

Hoyt examined the frictional characteristics of λ-carrageenan and sodium carrageenan [91]. He reported a high drag reduction efficiency of more than 60% at 2000 ppm carrageenan in pipe flow, and proposed that carrageenan extracted from commercial preparations has good drag-reducing characteristics [92].

**Figure 14:**
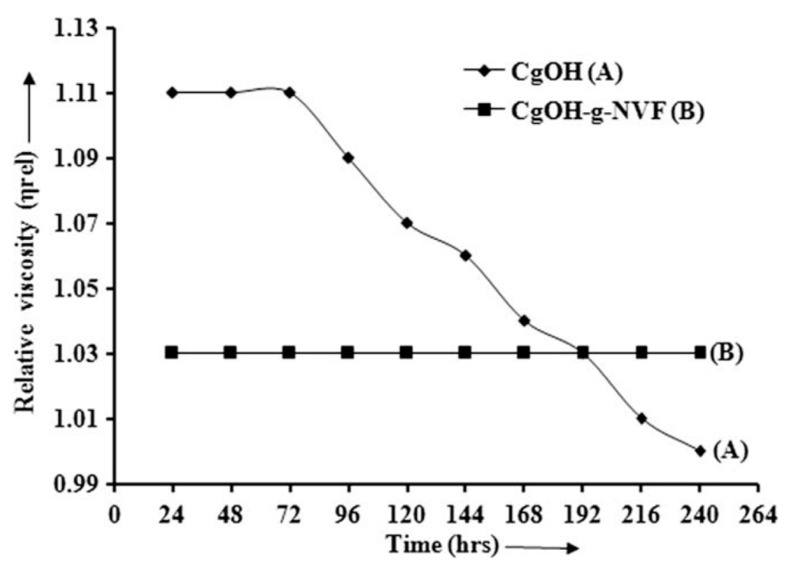
Resistance to the biodegradability of κ-carrageenan and its graft copolymer (κ-carragenan-*g*-*N*-vinyl formamide), reprinted with permission from [93]. Copyright Elsevier, 2010.

Recently, to improve its ability to resist biodegradation, many studies examined advanced carrageenan-based materials. Mishra et al. [93] adopted κ-carrageenan and another effective drag-reducing agent, poly(*N*-vinyl-formamide) (PNVF), to carry out a copolymerization reaction. The resistance to the biodegradability of kappa-carrageenan and synthesized κ-carrageenan-*g*-*N*-vinyl formamide was compared in a NaNO_3_ solution for 10 days, as shown in Figure 14. The graft copolymer initially showed a lower relative viscosity than kappa-carrageenan, which was maintained for up to 10 days, indicating the strong ability of resistance to biodegradation. In addition, they also suggested good water swelling ability, enhanced drag reduction effectiveness, and flocculation efficiency of graft copolymer (κ-carrageenan-*g*-*N*-vinyl formamide), which was expected to be used in the treatment of coal wastewater. Similarly, the enhanced drag reduction effectiveness and minimized biodegradation can also be obtained by the graft copolymer (κ-carrageenan-*g*-vinylsulfonic acid) synthesized by Yadav et al. [94].

### 4.6. Okra Mucilage

**Figure 15:**
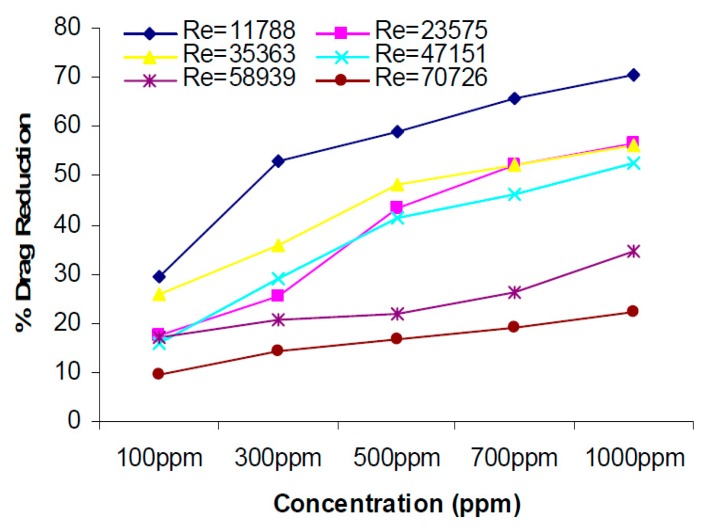
Effect of concentration on the percentage drag reduction for Okra-Natural Mucilage dissolved in water flowing through 0.015 m ID pipe, reprinted with permission from [95]. Copyright Centre for Graduate Studies, Universiti Malaysia Pahang, 2009.

Okra is a flowering plant from the mallow family. The okra mucilage obtained from the okra pod is a polysaccharide consisting of d-galactose, I-rhamnose and galacturonid acid. As a high molecular weight polymer, okra mucilage has been evaluated as a drag-reducing agent. Ahmad et al. [95] examined the effect of the addition of okra natural mucilage as a drag-reducing agent in a closed loop of water flow system with different flow rates and different pipe diameters. Figure 15 shows that the drag reduction percentage increases with increasing concentration of okra natural mucilage, and a maximum drag reduction percentage of 71% was obtained using 1000 ppm of additive in a 0.015 m ID pipe diameter. 

Abdulbari et al. [96] also evaluated okra mucilage and concluded that the biodegradable, highly effective, and low cost of okra mucilage give it an advantage for use as a drag-reducing agent in water transportation.

Subsequently, Abdulbari et al. [97] examined the drag reduction performance of okra mucilage-acrylonitrile grafted polymer in water and hydrocarbon liquid, respectively. This test was conducted in a closed-loop system and its drag reduction performance increased with increasing fluid velocity, pipe length, and internal pipe diameter. The highest drag reduction of 60% was obtained using 1000 ppm of grafted okra mucilage.

**Figure 16:**
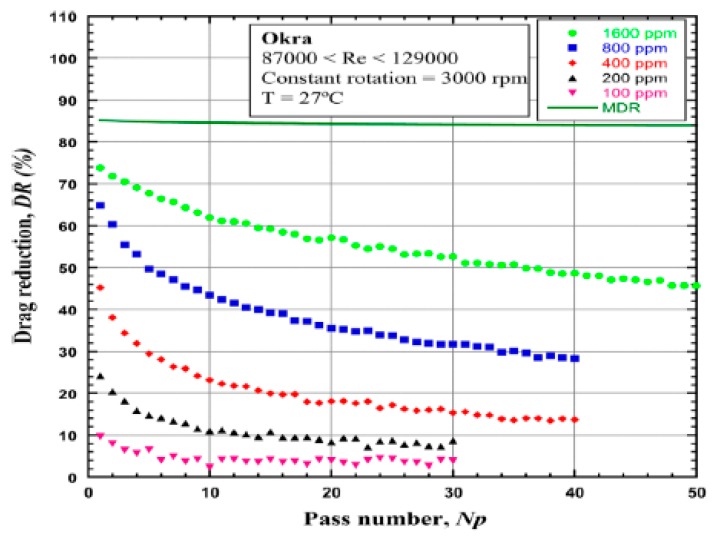
Drag reduction as a function of the number of passes through the system for a range of concentrations), reprinted with permission from [98]. Copyright Springer, 2016.

Recently, Coelho et al. [98] analyzed the capability of okra mucilage and fiber as drag reducers with high Reynolds number flows in a pipeline. Figure 16 shows that the maximum drag reduction was achieved using 1600 ppm of polymeric additive, which is close to the maximum drag reduction (MDR) asymptote. They attributed the drag reduction to de-aggregation rather than to mechanical degradation because of the loss of efficiency, which is similar to the rigid materials, such as xanthan gum and guar gum. Degradation is only perceptible after 24 h. Therefore, they considered that okra mucilage can be a substitute for synthetic polymers or other biopolymers owing to its low cost and availability.

In addition to the above, many other bio-polymers have been assessed as potential drag-reducing agents. Examples include aloe vera, chitosan, and latex. Abdulbari et al. [99] examined the drag reduction performance of aloe vera using a closed-loop liquid circulation system and found that aloe vera is an effective natural drag-reducing agent; a maximum drag reduction percentage of 63% was achieved by the addition of 400 ppm mucilage in turbulent flow.

Chitosan, a linear polysaccharide, was also assessed as a drag-reducing agent in aqueous systems. Abdulbari et al. [100] synthesized a graft copolymer of chitosan with acrylamide and examined the effects of the chitosan solution concentration on the drag reduction performance. The drag reduction percentage increased with increasing concentration. Moreover, a maximum drag reduction of 80.42% was obtained at 300 ppm of graft copolymer in a water flowing system. 

Table 1 summarizes the drag reduction efficiencies of the above natural biopolymers.

## 5. Applications

Since the discovery of the polymer-induced drag reduction phenomenon, their use has been technically and economically attractive for many areas, such as crude oil transportation, irrigation, hydro-transport, sewers, fire-fighting system, biomedical areas, oil well, heating circuits, and marine fields. Among them, some applications, such as fire-fighting systems and oil wells have been implemented, whereas others are still in the pilot phase. This paper focuses on several important applications of bio-based polymer drag-reducing agents.

### 5.1. Petroleum Industry

The petroleum industry is one of the main applications of drag-reducing agents. Both synthetic polymers and bio-polymers are used widely in the petroleum industries, such as hydraulic fracturing, drilling fluid [101], crude oil transportation [102,103], and enhanced oil recovery [104,105]. Commercially, gums are added as viscosifying agents to a fracturing fluid. In the fracturing process, the thickened liquid can carry drill cuttings into fractured rock to produce a path for oil to flow to the well bore [106]. Singh et al. [107] examined the rheological and thermal characteristics of guar gum-polystyrene copolymer, and suggested the potential use of the synthesized copolymer in the petroleum industry and mineral processing. Recently, a number of bio-based drag-reducing agents related to the applications of drilling fluids have been published, such as oxidized guar [108], carboxymethyl guar [101], and xanthan heterpolysaccharide biopolymers [109].

### 5.2. Biomedical Application

The biomedical area, as another major application for bio-based drag-reducing agents, has been assessed for several decades. The effects of drag-reducing polymers on tissue perfusion and the hydrodynamics of blood flow in animals has been demonstrated. Sakai et al. [110] adopted aloe-based drag-reducing agents in an animal model of acute myocardial ischemia, and reported a decrease in animal mortality. Kameneva et al. [111] evaluated the effects of small-volume resuscitation with drag-reducing agents in a rat hemorrhage model, and confirmed the hypothesis that resuscitation with a small quantity of drag-reducing agents prolongs survival in rats with lethal hemorrhagic shock. Many studies have examined the effects of biocompatible polymeric drag reduction additives on reducing atherosclerosis [112] and increasing blood flow [113]. A drag reducing polymer can be used as an effective tool for the prevention or treatment of circulatory diseases.

### 5.3. Flocculants

Flocculants are essential in many industrial processes that separate solid particles from aqueous suspensions. Polymeric flocculants in water and industrial effluent treatment have attracted considerable attention owing to their low doses, easy handing, and insensitivity to pH. Many synthetic polymers, such as PAAM, PAA, and poly(diallyl dimethyl ammonium chloride) as well as biopolymers of starch, gum, and alginic acid have been used as retention aids or flocculants. With continuous research and development, many grafted polysaccharides have been assessed extensively as efficient flocculating agents and turbulent drag reducers for a range of industrial applications. Stelter et al. [85] compared the flocculating efficiency of grafted and ungrafted amylopectin with PAM. They reported that the grafted amylopectin exhibited enhanced flocculation. Similarly, Singh et al. [114] grafted PAM branches into the backbone of polysaccharides, including GG, XG, CMC, and starch. They suggested the possibility of developing efficient, shear-stable and biodegradable flocculants. In addition, some novel flocculants, such as sodium alginate-*g*-PAM [55] and κ-carrageenan-*g*-*N*-vinyl formamide [101], are still being explored.

### 5.4. Agricultural Application

As bio-based drag-reducing agents are harmless to plants and soil, turbulent drag reduction phenomena are used widely in the agricultural field, particularly in sprinkler irrigation. Many polymers, such as PEO, CMC, PAAM, XG, and GG, are often used for various sprinkler irrigation experiments. Singh et al. [114] conducted extensive experiments, and demonstrated that the addition of drag-reducing agents reduced the energy requirements and increased the coverage area. The solvation of additives and the increased elongational viscosity of solutions reduce the percolation loss of water in the soil. Singh et al. [115] also prepared a slow release urea by mixing urea with GG to enhance the utilization of urea. Recently, Phukan et al. [77] carried out irrigation system experiments with different concentrations of commercial GG and purified GG along with different injection methods. They suggested that purified GG has enhanced drag reduction properties, and that the homogeneity of the polymer solution has an effect on the drag reduction property. These studies suggested that the application of drag-reducing agents is of great significance to the high efficiency of agricultural production, particularly for water-stressed countries.

## 6. Conclusions

This paper reviewed the state-of-art work on bio-based polymers, including xanthan gum, guar gum, DNA, amylopectin, cellulose, carrageenan, and okra mucilage, as promising drag-reducing agents, along with their drag reduction characteristics and applications. The drag-reducing abilities of these bio-polymers in aqueous media show that these bio-polymers can be used to replace artificial polymers to solve safety and environmental issues. Future work could be focused on identifying new bio-based drag-reducing agents and improving the drag reduction efficiency of existing bio-polymers through chemical modifications. In addition, the solubility of bio-polymers can be assessed for possible extension to applications in other non-aqueous systems. These bio-polymers are expected to be the preferred choice for a wider range of applications.

## Figures and Tables

**Table 1:** Drag reduction performance of bio-polymer additives.

Biopolymer	Solvent	Concentration (ppm)	Max *DR*%
Xanthan gum [66]	Water	200	36.2
Xanthan gum [66]	Water and KCl	200	34
Xanthan gum [62]	Water and NaCl	200	33
Guar gum [71]	Water	200	32
λ-DNA [79]	Water	2.7	19.8
CT-DNA [84]	Water	20.25	5.8
Amylopectin [87]	Water	30	27.3
Hydroxyethyl cellulose [88]	Water	300	42
Carboxymethyl cellulose [89]	Water	1000	42
Carrageenan [93]	Water	2000	60
Okra [95]	Water	1000	71
Aloe vera [99]	Water	400	63
Chitosan [100]	Water	300	80.4
